# Molecular events in MSC exosome mediated cytoprotection in cardiomyocytes

**DOI:** 10.1038/s41598-019-55694-7

**Published:** 2019-12-17

**Authors:** Rajshekhar A. Kore, Jeffrey C. Henson, Rabab N. Hamzah, Robert J. Griffin, Alan J. Tackett, Zufeng Ding, Jawahar L. Mehta

**Affiliations:** 1 Department of Medicine, Cardiology Division, University of Arkansas for Medical Sciences, Little Rock, AR and the Central Arkansas Veterans Healthcare system, Little Rock, AR 72205 USA; 2 Department of Radiation Oncology, University of Arkansas for Medical Sciences, Little Rock, AR 72205 USA; 3 Department of Biochemistry and Molecular biology, University of Arkansas for Medical Sciences, Little Rock, AR 72205 USA; 4 Center for Integrative Nanotechnology Sciences, University of Arkansas at Little Rock, Little Rock, AR 72204 USA

**Keywords:** Apoptosis, Heart failure

## Abstract

A host of hormonal-metabolic alterations take place following exposure of cardiomyocytes to hypoxia and other noxious stimuli. Here, we demonstrate that exposure of cultured rat cardiomyocytes to lipopolysaccharide (LPS) resulted in upregulation (~1.5 fold) of oxidized low-density lipoprotein receptor-1 (LOX-1). There was also a marked increase in apoptosis 12 hrs after LPS treatment with caspase-3 levels being significantly elevated (~1.3 fold) and a significant increase in LDH release at 24 hrs. Interestingly, there was a ~1.4-fold upregulation of LC-3 expression post-LPS treatment indicating development of autophagy, which probably is a compensatory response to combat cellular injury induced by LPS. Treatment with LPS also reduced the size and morphology of cardiomyocyte spheroids. In an attempt to limit LPS-induced injury, cardiomyocytes were treated with exosomes derived from mesenchymal stromal cells (MSCs). We noted a significant suppression of LOX-1 expression that in turn suppressed apoptosis as well as autophagic response and restored spheroid morphology. Mass spectrophotometric analysis of MSC exosomes revealed a cargo rich in proteins which are involved in pathways negatively modulating cell death and apoptosis while promoting cell survival. This is first report to our knowledge on the initial molecular events in MSC exosome mediated cytoprotection of stressed cardiomyocytes.

## Introduction

Myocardial infarction (MI) is a leading cause of mortality worldwide^1^. Wide-spread apoptosis occurs in the tissue areas surrounding the infarct following MI. Involvement of autophagy in acute and chronic ischemia is also well documented^2,3^. The importance of autophagy in limiting the extent of eventual injury has been recently demonstrated in a number of *in vitro* and *in vivo* studies. It has been reported that preceding starvation (a basic stimulant of autophagy) and intermittent reperfusion post-MI significantly reduces infarct size^4,5^ Reports have shown that autophagy during myocardial ischemia is a major determinant of tissue survival^6,7^.

Lectin-like oxidized LDL receptor-1 (LOX-1) is a type C receptor for ox-LDL which is upregulated by angiotensin II and pro-inflammatory cytokines^8–11^. Under physiological conditions, expression of LOX-1 in cardiomyocytes is low, but it is highly inducible when these cells are subjected to various forms of metabolic, inflammatory, ischemic, oxidative and mechanical stress. Expression of LOX-1 appears to be important in the eventual determination of extent of ischemic myocardial injury^3,12–14^, since abrogation of LOX-1 reduces infarct size, number of apoptotic cells, inflammatory signaling, ROS generation and cardiac function. Recent studies show that exposure of cardiomyocytes to stress also induces signals for autophagy^15^. The autophagy signals appear early and are short-lasting, where signals for apoptosis are relatively late in onset, but persist for a long duration^3^.

Attempts to exploit stem cells in hopes of regenerating/repairing cardiac muscle and improving cardiac function^16–19^, have so far revealed only limited degree of success^20–22^. A major limitation in these studies is inability of stem cells to respond and adapt favorably to the damaged microenvironment. Use of stem cell exosomes offers a promising novel approach for post-MI intervention to decrease scar formation and fibrosis, and help regenerate cardiac muscle^23–27^. Exosomes, a distinct class of constitutively secreted vesicles of endosomal origin, carry precious cargo of proteins and genetic material. They possess the ability to alter the transcriptome and proteome of the recipient cell, modulating pathways governing apoptosis, cell growth, proliferation and differentiation. In place of pluripotent or multipotent stem cells, which are unfavorably affected by signals from the damaged cardiac tissue microenvironment, treatment with stimulated MSC exosomes will render the ischemic heart resistant to micro-environmental stressors and help contain spread of ischemic damage^28^. Exosomes from various stem cell sources have been employed to provide protection against ischemic damage. However, we decided to focus our efforts on the use of MSC exosomes for the stem cells can potentially be readily harvested from MI patients without any need for differentiation using differentiating factors and also the fact that they are readily available. The primary aim of our study was to decipher molecular events in cardiomyocytes exposed to lipopolysaccharide (LPS), with particular reference to the expression of LOX-1, and signals for apoptosis and autophagy. Further, we aimed to determine if MSC exosome treatment would provide cytoprotection by blocking these early events. Lastly, we conducted proteomics to determine pathways of LPS-mediated cellular injury and their modulation by MSC exosomes.

## Results

### Characterization of exosomes

**Figure 1:**
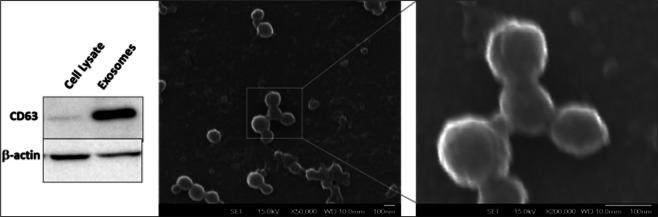
Characterization of exosomes. Western blots show enrichment of CD63 in exosomes while it is barely detected in whole cell lysates. Exosomes were plated onto a poly-L-ornithine coated glass slide and SEM images were acquired. The exosomes show a uniform size distribution of around 80 nm. Scale bar = 100 nm.

Scanning electron microscopy (SEM) analysis of suspended exosome pellet was used to confirm that the ultracentrifugation pellet isolated from MSC cultures consisted of discrete vesicles and not parts of damaged intracellular cellular organelles or membranes. Nanoparticle tracking analysis of exosomes revealed an average diameter of 118 nm (Supplement Fig. 1). Probing for exosomal marker, Western blot of equivalent quantities of proteins show enrichment of CD63 in the exosome sample compared to whole cell lysate. High magnification scanning electron microscopy micro pictographs obtained using an 80 keV electron beam showed presence of vesicles of uniform size and diameter (Fig. 1). Mass spectrometry analysis revealed the absence of several organelle markers such as endoplasmic reticulum marker calnexin, golgi marker GM130, mitochondrial marker proteins such as cytochrome C oxidase, ABCD3, ATP5A1 etc. MS analysis revealed the presence of several exosomal markers such as CD63, CD9 and CD81 (Supplementary Results). These results along with electron micropictographs indicate that the pellets isolated from conditioned media of MSC cultures by sequential centrifugation constitute a distinct set of secretory vesicles.

### Injurious effect of LPS on cardiomyocytes and rescue by MSC exosomes

**Figure 2:**
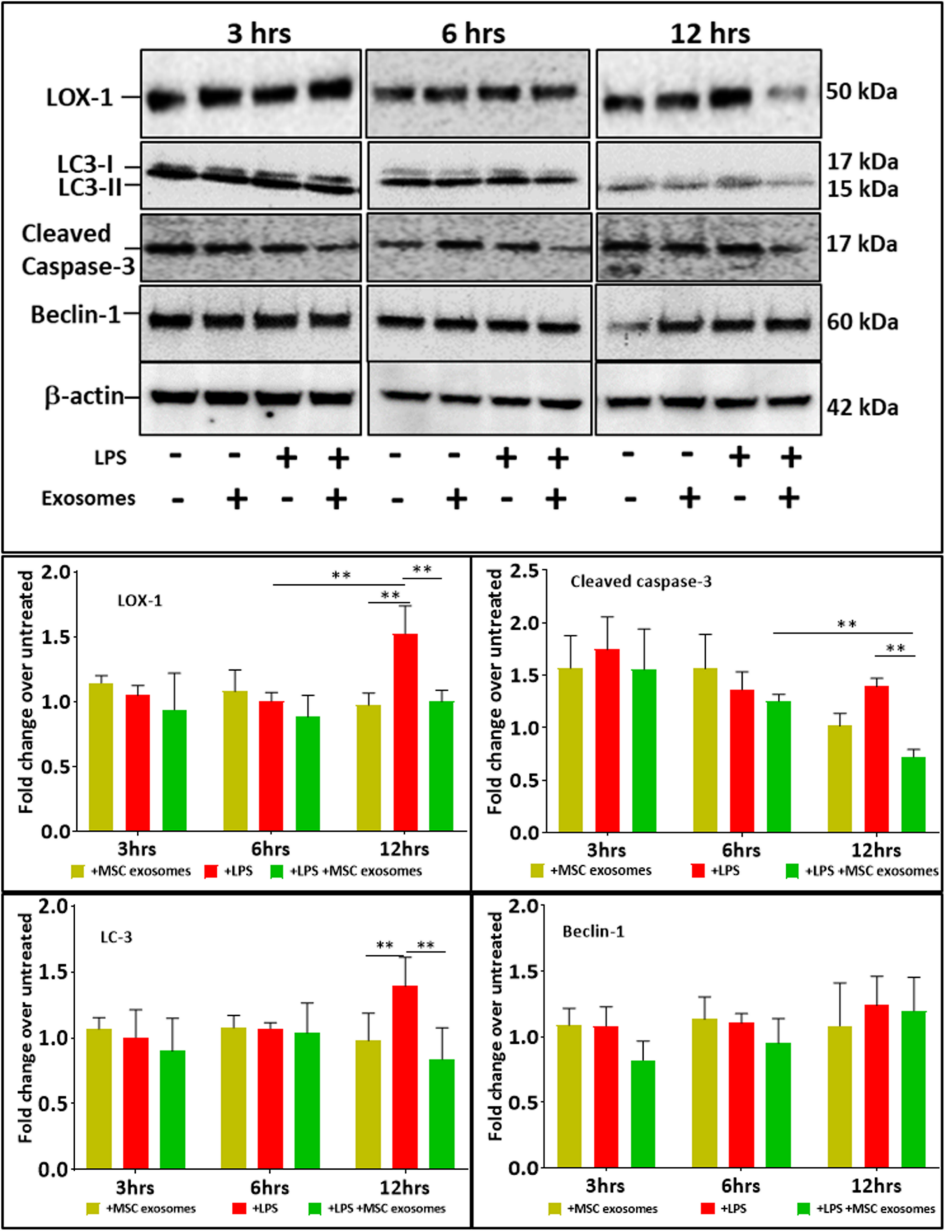
Monolayer cultures of cardiomyocytes were exposed to LPS (100 ng/ml) for 1 hour and then treated with MSC exosomes. LPS induced LOX-1 expression at 12 hrs of exposure. LPS also induced apoptosis (cleaved Caspase-3) and autophagy (LC3). Treatment with MSC exosomes decreased LOX-1, cleaved caspase-3 and LC-3 levels in cardiomyocytes stressed with LPS over a period of 6–12 hrs, but had no effect on expression of Beclin-1. LOX-1 levels were decreased with MSC exosomes. Data in mean ± SD, n = 4 *p < 0.05, vs LPS treatment based on 3 independent experiments. Full-length blots are presented in Supplementary Fig. 2.

**Figure 3:**
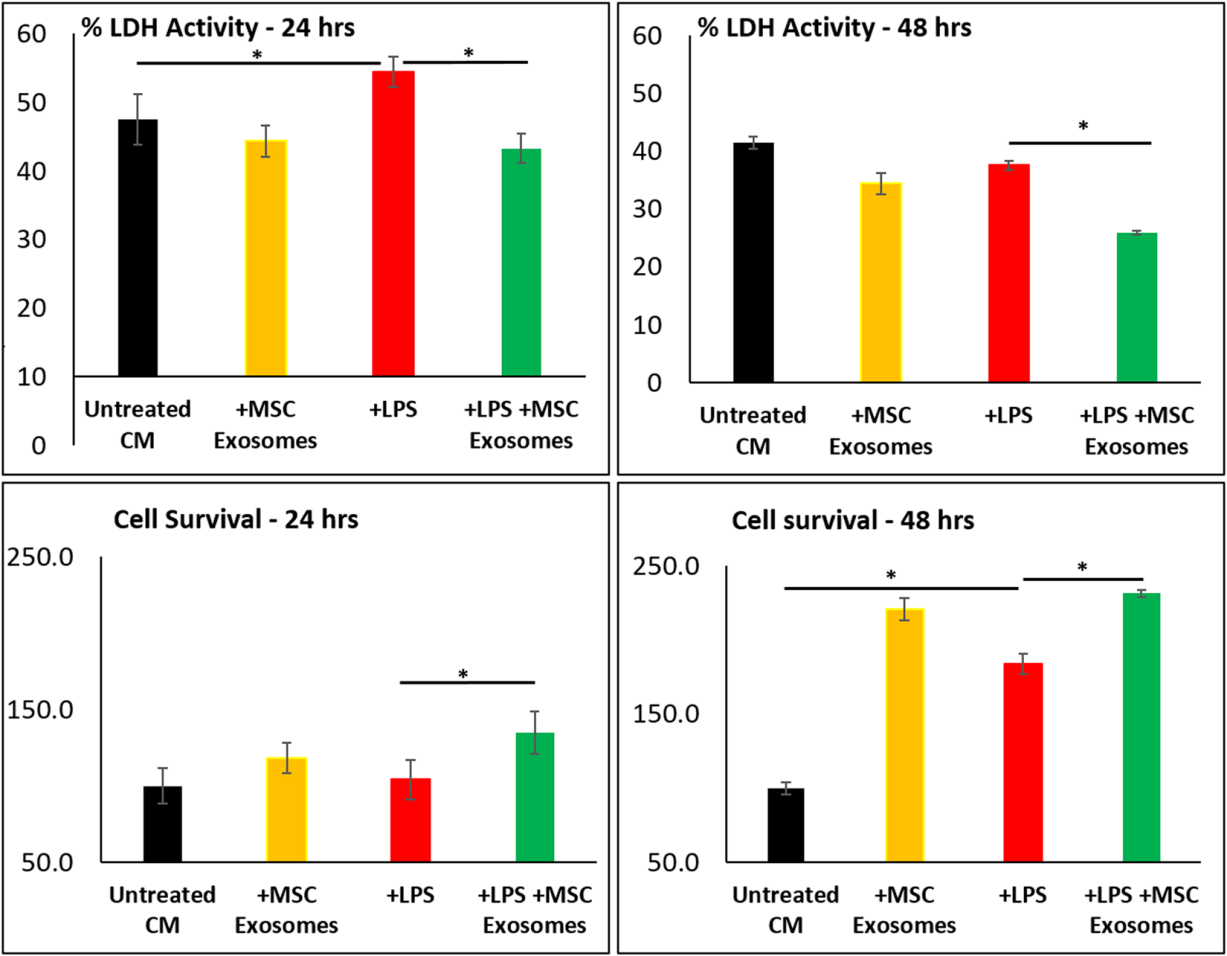
Cell death (LDH release) and cell survival assays on cardiomyocytes. Cells were first treated with LPS (100 ng/ml) for 1 hr and then treated with MSC exosomes in presence of LPS. Treatment with MSC exosomes led to decrease in cell death and increase in cell survivability. Data as mean ± SD, n = 4. *p < 0.05 vs LPS treatment based on 3 independent experiments.

LOX-1 expression was significantly upregulated in cardiomyocytes early, at 12 hr after exposure to LPS (100 ng/ml) (Fig. 2) which correlates with our previously published data^13,29^. We measured occurrence of apoptosis and autophagy in response to LPS (Fig. 2). We noted a marked increase in apoptosis (expression of Caspase-3) as well as autophagy signal LC3-II and Beclin-2. Of note, while the autophagy marker LC3-II was significantly elevated (≈1.4 fold), Beclin-1 expression over the same time period also increased (≈1.2 fold), but not significantly. LDH activity as a marker of cell death slightly increased at 24 hr. Interestingly cell survival measured as MTT assay also increased under LPS stress, especially at 48 hrs (Fig. 3).

Most interestingly, treatment of cardiomyocytes with MSC exosomes in presence of LPS induced stress (1 hr after) rescued cells from LOX-1 expression, and occurrence of apoptosis and autophagy (Fig. 2). Treatment of cardiomyocytes with MSC exosomes also protected cardiomyocytes from death (LDH release) (P < 0.05). Notably MSC exosome treatment further increased cell survival at both 24 and 48 hr time points. Importantly, MSC exosomes alone had no effect on LOX-1 expression, occurrence of apoptosis and autophagy signals (Fig. 2). MSC exosomes, however, enhanced cardiomyocyte survival (Fig. 3).

### Effect of exosomes on Cardiomyocyte spheroids

**Figure 4:**
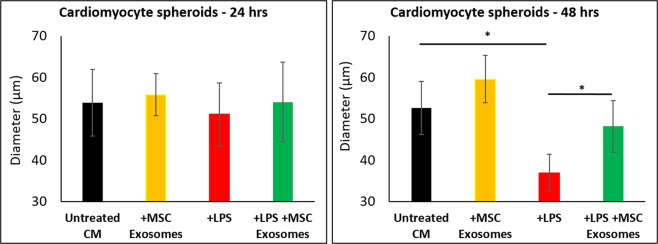
Cardiomyocyte spheroids were exposed to LPS (100 ng/ml) and then treated with MSC exosomes. Exposure to LPS reduced the spheroid diameter by >50% at 48 hrs. Treatment with exosomes rescued cardiomyocyte spheroid morphology in presence of LPS. *p < 0.05 vs LPS treatment based on 4 independent experiments.

We wondered if LPS and exosomes would have any effect on tissue morphology. While monolayer cell cultures present a reductionist approach to study cellular mechanisms in response to certain stimuli, spheroid cultures accurately mimic natural physiological responses and cell-to-cell interactions^30,31^. Using the hanging drop method, cardiomyocytes were cultured as spheroids. Cardiomyocyte spheroids were stressed with LPS and then treated with MSC exosomes. While LPS significantly decreased cardiomyocyte spheroid diameters progressively over the 48 hr period, MSC exosomes rescued spheroid integrity over the same time point (Fig. 4). (Representative images of the spheroids are shown in Supplement Fig. 7).

### Proteomic analysis of MSC exosomes

**Figure 5:**
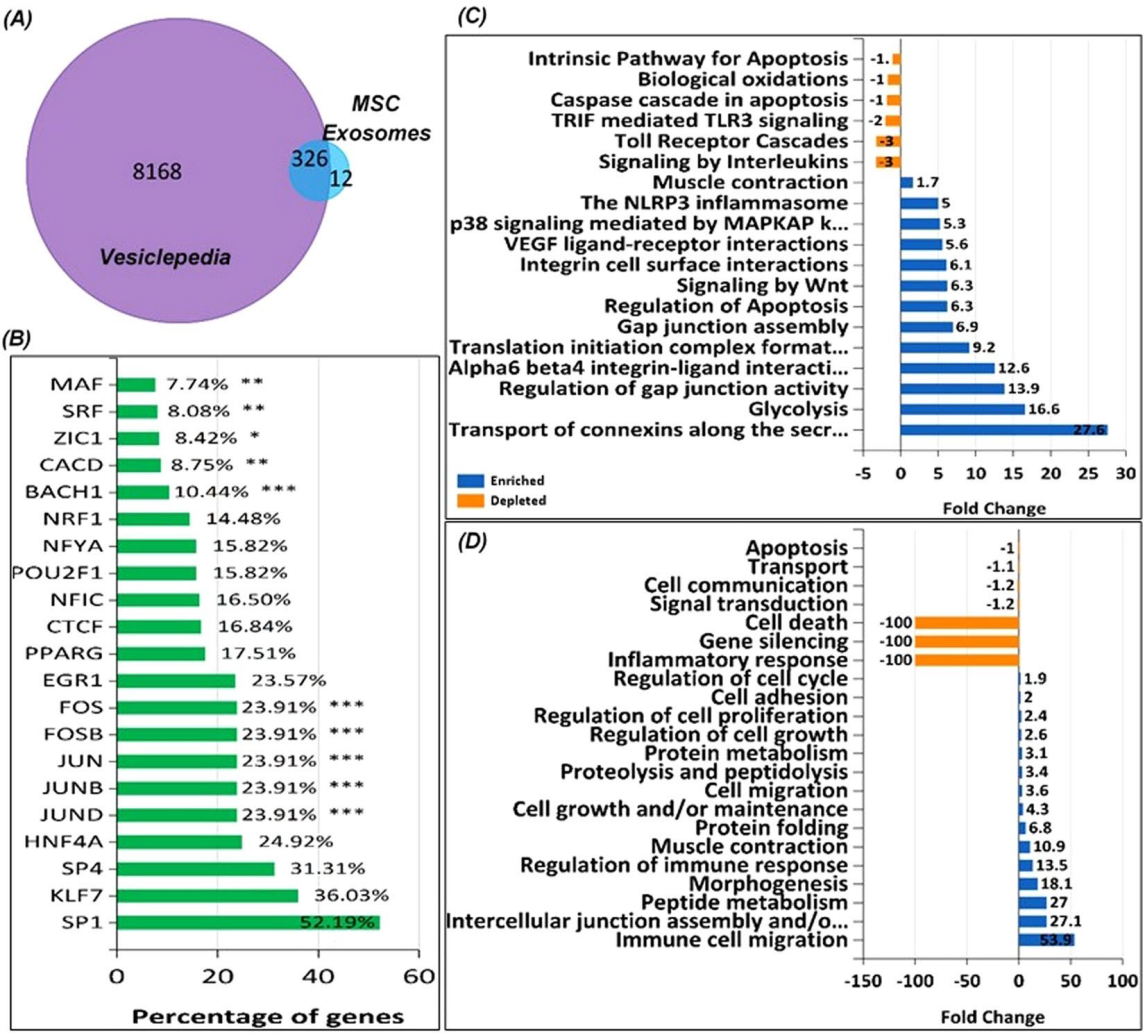
Compared to the vesicle secreted protein database, MS analysis using FunRich^32^ analysis tool identified a number of similar and unique proteins secreted in MSC exosomes (**A**). Proteomic analysis identified different transcription factors in MSC exosomes (**B**). FunRich analysis revealed the different pathways which the secreted proteins modulate (**C**) and the processes which the exosomal proteins regulate (**D**). IPA analysis revealed the many this exosomal cargo modulates cellular proliferation, cell survival, and in recipient cells.

Proteomic analysis identified a host of proteins (≈338) secreted in MSC exosomes. Using FunRich^32^ analysis for extracellular vesicle (EV) proteins secreted by all cell types available in the database, we identified 326 common classes of proteins enriched in EV subsets (Fig. 5A) and 12 unique to the MSC exosomes. Among the entire set of proteins, we identified various transcription factors (TFs) such as SP1, KLF7, SP4, JUN, JUND, JUNB, FOSB and FOS which are involved in regulating expression of their target genes in recipient cells (Fig. 5B). Other proteins secreted via exosomes shown in Fig. 5C,D differentially modulate various pathways and are involved in processes such as apoptosis, protein metabolism, cell growth and proliferation. In addition, analysis of exosomal proteins using Integrated Pathway Analysis (IPA Qiagen Bioinformatics)^33^ software showed that some of the proteins are involved in upregulating pathways governing proliferation of immune cells, muscle formation and function and angiogenesis (Supplement Fig. 3A) while others are involved in downregulating processes involved in infarct size, organ degeneration, cytolysis and hypersensitive reactions (Supplement Fig. 3B).

### Proteomic analysis of cardiomyocytes treated with LPS followed by exosomes

We conducted proteomic analysis of cardiomyocytes stimulated with LPS and then rescued with MSC exosomes. As expected, compared to untreated cardiomyocytes, LPS treatment changed the proteome (1580 identified proteins) of these cardiomyocytes (Supplement Fig. 3A) with many proteins being differentially expressed; for example, PSME4, ACADVL, FAM91A1, GNA11, STAT6 (downregulated) and NCBP1, WBP11, ACAT2, FXR2, SPART (upregulated) (Supplement Fig. 4B,C). Many of the proteins identified are related to the upregulation of apoptotic process, response to oxidative stress, regulation of cell death and myoblast migration, while proteins related to fatty acid catabolism, response to cytokine stimulus, cardiac conduction, DNA replication proof reading and regulation of cardiac conduction were downregulated. (Supplement Fig. 4D).

**Figure 6:**
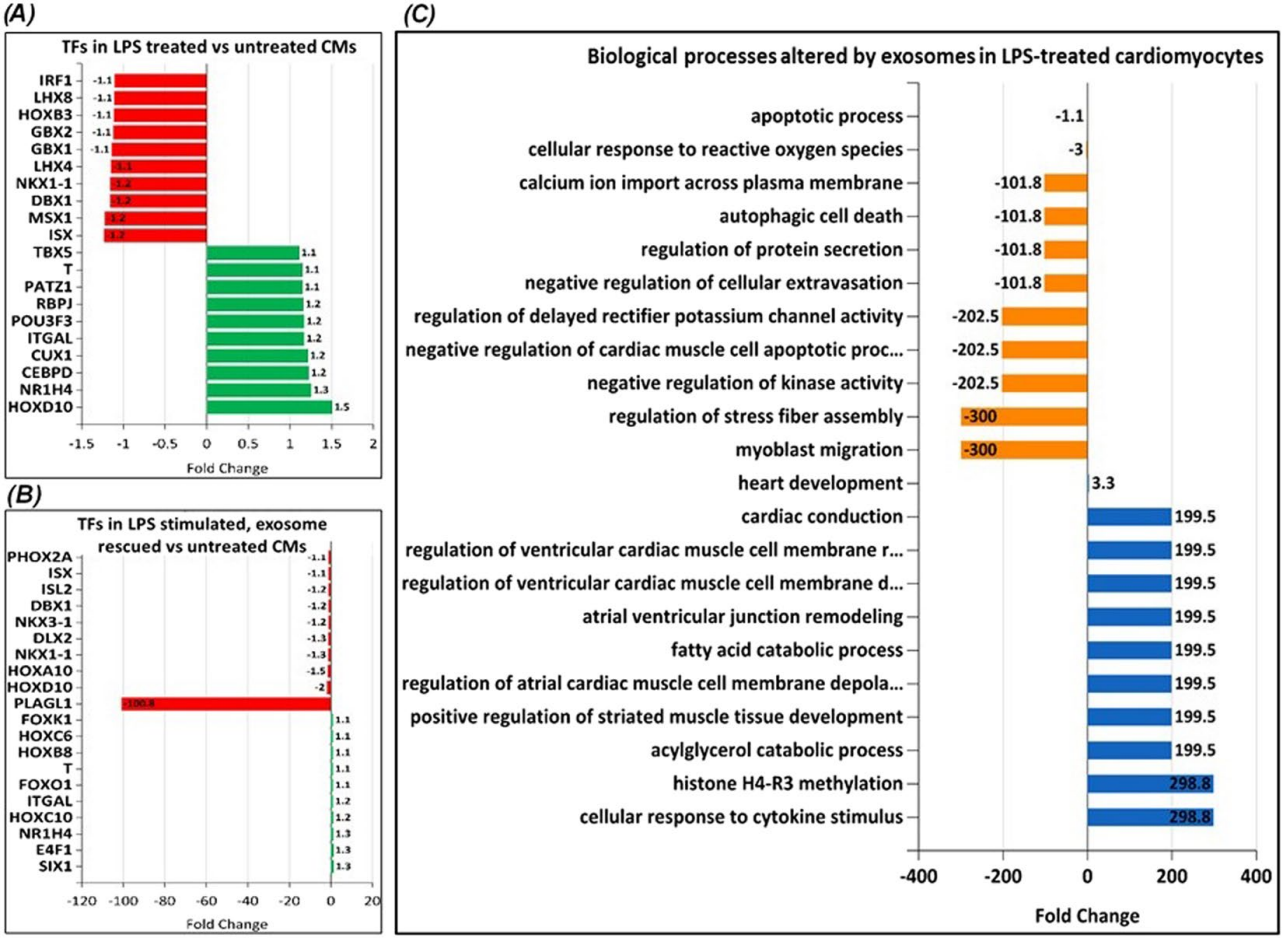
MS analysis of cardiomyocytes (CMs) using FunRich^32^ analysis tool: Differences in response to exosome treatment in cells can be attributed to the functional exosomal cargo in regulating gene expression such as changes in (**A**) expression of transcription factors (TFs) in LPS stressed cells or (**B**) LPS treated cells rescued by MSC exosomes. (**C**) Analysis of biological processes of proteins identified by MS in exosome rescued cardiomyocytes post LPS treatment. The processes presented are in comparison against LPS stimulated cardiomyocytes.

**Figure 7:**
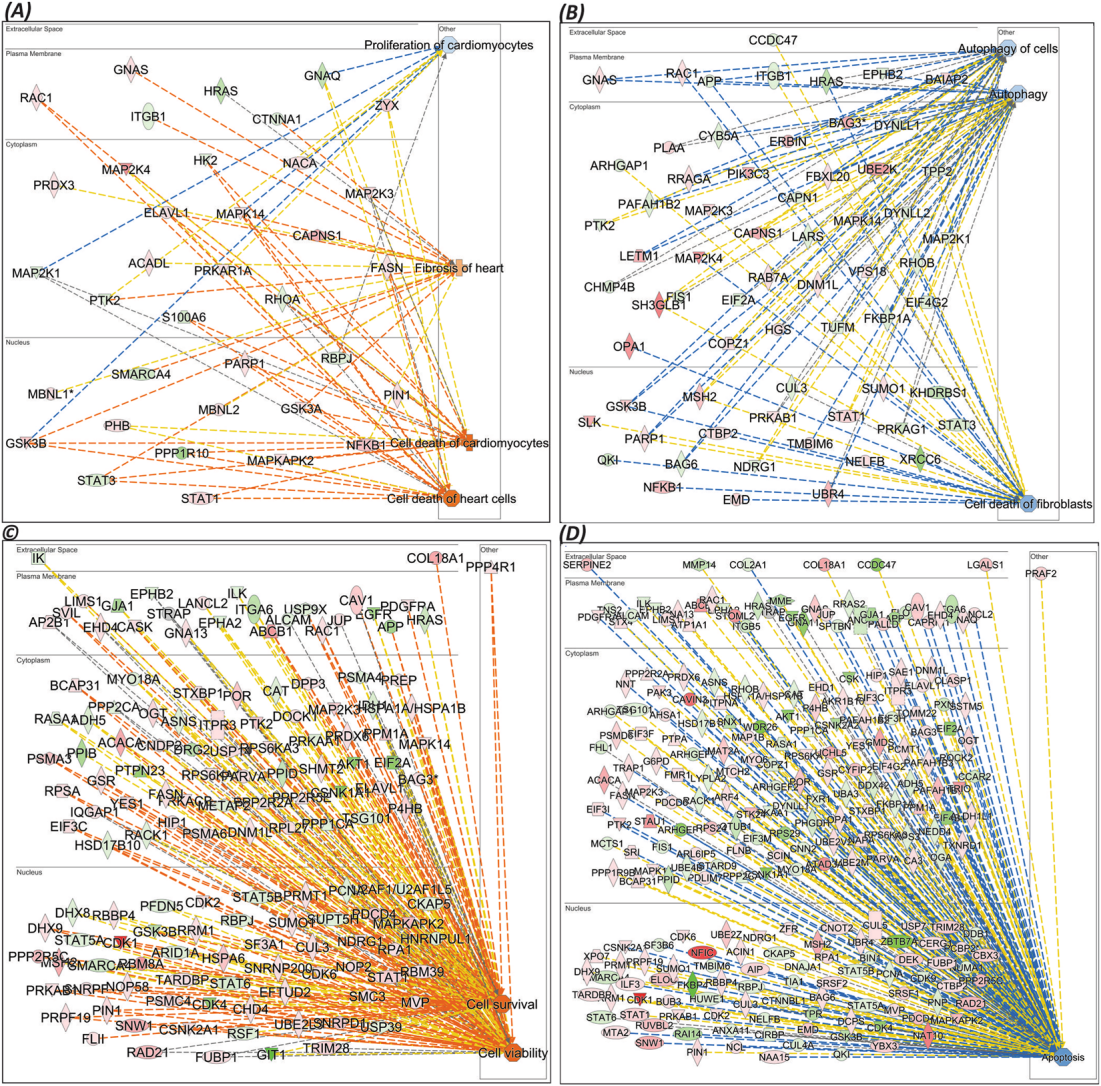
Network analysis: IPA (QIAGEN Inc., https://www.qiagenbioinformatics.com/products/ingenuity-pathway-analysis)^33^ network analysis revealed upregulated signaling networks leading to cardiomyocytes death and cardiac fibrosis (**A**) and suppressed pathways leading to proliferation of cardiomyocytes, autophagy and fibroblast cell death (**B**). Exosome treatment of LPS stimulated cells upregulated signaling networks leading to increased cell viability and cell survival (**C**) while it decreased apoptotic signaling network (**D**).

Many TFs were also found to be differentially expressed. Among the upregulated TFs were HOXD10, NR1H4, CEPBD, CUX1 and ITGAL, while ISX, MSX1, DBX1, LHX4 and GBX1 were downregulated (Fig. 6A). IPA^33^ network analysis revealed upregulated signaling networks leading to cardiomyocytes death and cardiac fibrosis (Fig. 7A) and suppressed pathways leading to proliferation of cardiomyocytes, autophagy and fibroblast cell death (Fig. 7B).

Further, treatment with MSC exosomes of LPS stimulated CMs revealed expression of different sets of proteins. For example, exosome treatment of LPS stimulated CMs showed expression of 1587 identified proteins (Supplement Fig. 5A) among which GIT2, GIT1, VPS29 and DNAJA2 were downregulated, and CDK1, MACF1, ACAT2 were CAND2 were upregulated (Supplement Fig. 5B,C).

Alterations in TFs are shown in (Fig. 6B). The most prominent changes were seen in expression of SIX1, E4F1, NR1H4, HOXC10 and ITGAL(upregulated), and expression of PLAGL1, HOXD10, HOXA10, NKX1-1 and DLX2 (downregulated). Surprisingly, compared to LPS treated cells, the level of one transcription factor, PLAGL1 was dramatically modulated when LPS stimulated cells were treated with MSC exosomes (Fig. 6B). Most importantly, treatment of LPS stimulated cells with exosomes caused significant downregulation of cellular processes such as apoptosis, oxidative stress, autophagic cell death and myoblast migration and upregulation of processes such as fatty acid catabolism, response to cytokine stimulus, cardiac conduction and regulation of cardiac muscle membrane depolarization and repolarization (Fig. 6C) which is in stark contrast to those seen with LPS stimulated cells prior to treatment with exosomes (Supplement Fig. 3D). IPA network analysis showed exosome treatment of LPS stimulated cells upregulated signaling networks leading to increased cell viability and cell survival (Fig. 7C) while it decreased apoptotic signaling network (Fig. 7D).

## Discussion

While various surgical and pharmacotherapeutic interventions have significantly improved patient outcomes following AMI, there is a major need for additional therapeutic options to repair or reverse damaged heart tissue post-MI. Attempts have been made to exploit stem cells by implanting them in the damaged cardiac tissue in hopes of regenerating/repairing cardiac muscle and improving cardiac function^16–19^. Results of these studies have so far revealed only limited degree of success^20–22^.

A promising novel area for recovery of various tissues from stress-induced injury is the use of stem cell exosomes^23,24,27^. Exosomes, a distinct class of constitutively secreted vesicles of endosomal origin, carry precious cargo of proteins and genetic material. Importantly, unlike stem cells, secreted exosomes do not respond to the damaged microenvironment but possess the ability to alter the transcriptome and proteome of the recipient cell, modulating pathways governing apoptosis, cell growth, proliferation and differentiation. Changes in exosomal cargo depend on cellular origin as well as the micro-environment in which the secreting cell finds itself in, reflecting the physiological state of the secreting cell^34,35^ Exosomal cargo secreted by cells plays a potent role in initiating transcription of target genes and translation of proteins which affect the phenotype of recipient cells. Importantly, unlike stem cells, secreted exosomes do not respond to the damaged microenvironment but possess the ability to alter the extracellular matrix, change the transcriptome and proteome of the recipient cell, modulating pathways governing apoptosis, cell growth, proliferation and differentiation.

Our previous work^36^ showed that LPS stimulates LOX-1 expression in cardiomyocytes. Increased LOX-1 expression induces oxidative stress and further drives the inflammatory pathways causing cell death. Ischemia to the heart caused by coronary artery ligation also induces LOX-1 expression^37,38^ The important role of LOX-1 in inducing myocardial injury became evident from studies wherein LOX-1 antibody or LOX-1 gene deletion dramatically reduced myocardial injury^37,38^. In other recent studies in cultured vascular smooth muscle cells, we showed that pro-inflammatory stimuli induced autophagy which was short-lasting and cellular apoptosis which was longer lasting^39^. In the present study, LPS indeed, significantly increased expression of LOX-1 in stimulated cardiomyocytes, which in turn led to increases in expression of proapoptotic caspase-3, and autophagy markers LC-3 and Beclin-1. However, when cardiomyocytes were first stimulated with LPS for one hour and then treated with MSC exosomes, we observed significant cytoprotective effects of MSC exosome treatment. MSC exosomes induced cytoprotective effects in cardiomyocytes by suppressing LOX-1 expression. Subsequently caspase-3 expression, and more importantly LC3 expression fell. Beclin-1 expression also increased with LPS treatment but not significantly. Previous studies have also shown discordance between LC3 and beclin-1 levels^40–42^. It is also possible that failure of beclin-1 to rise significantly is a type 2 error. We believe that occurrence of autophagy is a compensatory event to combat cellular injury caused by LPS^3,39^. These results collectively indicate that (I) LOX-1 is an early mediator of LPS-induced cardiomyocyte injury, and (II) MSC exosomes suppress LOX-1 expression and thus prevent LPS-mediated cell death. To our knowledge, these are the first set of studies showing a link between MSC exosome treatment and LOX-1 expression in cardiomyocytes which in turn affects cardiomyocyte autophagy and apoptosis.

To understand the function of exosomes, one needs to study the exosomal cargo and the influence this cargo has on recipient cells. We have previously demonstrated that changes in secreting cells’ microenvironment alters the exosomal cargo^34,35^. In the present investigation, we studied the proteome of MSC exosomes. Compared to all the proteins identified in secretory EVs by various tissues/cell types, we identified proteins in our samples which are unique to MSC exosomes (Fig. 5A). Among these of note were AOC1, SPARC, ARPC4-TTLL3, AFF1, THBS3, DCN and ACAN which are involved in cell-cell, cell-matrix attachment, modulation of extracellular matrix, acting polymerization while TNFRSF10D is known to inhibit TRAIL-induced apoptosis. Many of the identified exosomal proteins are involved in regulating or modulating a variety of biological pathways (Fig. 5C) in recipient cells such as downregulating intrinsic pathway for apoptosis, biological oxidation, and caspase cascade in apoptosis and toll-receptor cascades. These proteins positively regulate processes such as integrin cell surface interactions, glycolysis, MAPKAP kinase mediated p38 signaling, muscle contraction and gap junction assembly. Regulation of such pathways has a profound effect on processes such as cell death, gene silencing, inflammatory responses which are suppressed by exosomal proteins.

Further MS analysis revealed secretion of various transcription factors in exosomes. Transcription factors such as MAF, SRF, EGR1 and SP1 are involved in regulating transcription of genes which control differentiation, proliferation, metabolic pathways and cell survival. Exosomal SP1 has been implicated in providing significant protection against ischemia/reperfusion injury^43^. KLF7 regulates differentiation of progenitor cells and/or maintenance of phenotype cells^44^. Our functional assay data relative to improved cell survival and reduced cell death can be directly attributed to proteins carried in MSC exosomes.

Further, we investigated the changes in the proteome of cardiomyocytes stimulated with LPS and then rescued with MSC exosomes. We observed that MSC exosomes suppressed autophagic cell death, negatively regulated apoptotic processes, and suppressed cellular responses to reactive oxygen species, negatively regulated cellular extravasation and kinase activity. Processes such as cardiac conduction, fatty acid catabolism, regulation of striated muscle development, regulation of cardiac muscle repolarization and depolarization were positively regulated. The protective effects exhibited by MSC exosomes in LPS stimulated CMs are similar to those in cells treated with MSC exosomes alone (Supplement Fig. 6C–E). These data show the effect that MSC exosomes have on the expression of cytoprotective proteins in recipient cells.

We further analyzed the expression of cardiomyocyte transcription factors in response to LPS and further treatment with MSC exosomes. Compared to untreated cells, LPS alone induced expression of transcription factors such as HOX10, NR1H4, CEBPD, while suppressed expression of ISX, MSX1, DBX1, NKX1 and NOXB3 all of which control transcription of genes which negatively affect cardiac function and cell survival. Surprisingly, when LPS stimulated cardiomyocytes were treated with MSC exosomes, expression of one transcription factor, PLAGL1, was dramatically affected. PLAGL1 (pleomorphic adenoma gene-like 1) was also found to be significantly depleted in cells treated with MSC exosomes alone (Supplement Fig. 6B). PLAGL1 is a zinc-finger transcription factor whose target genes are involved in signaling, cell adhesion, extracellular matrix composition, and developmental disorders^45,46^.

**Figure 8:**
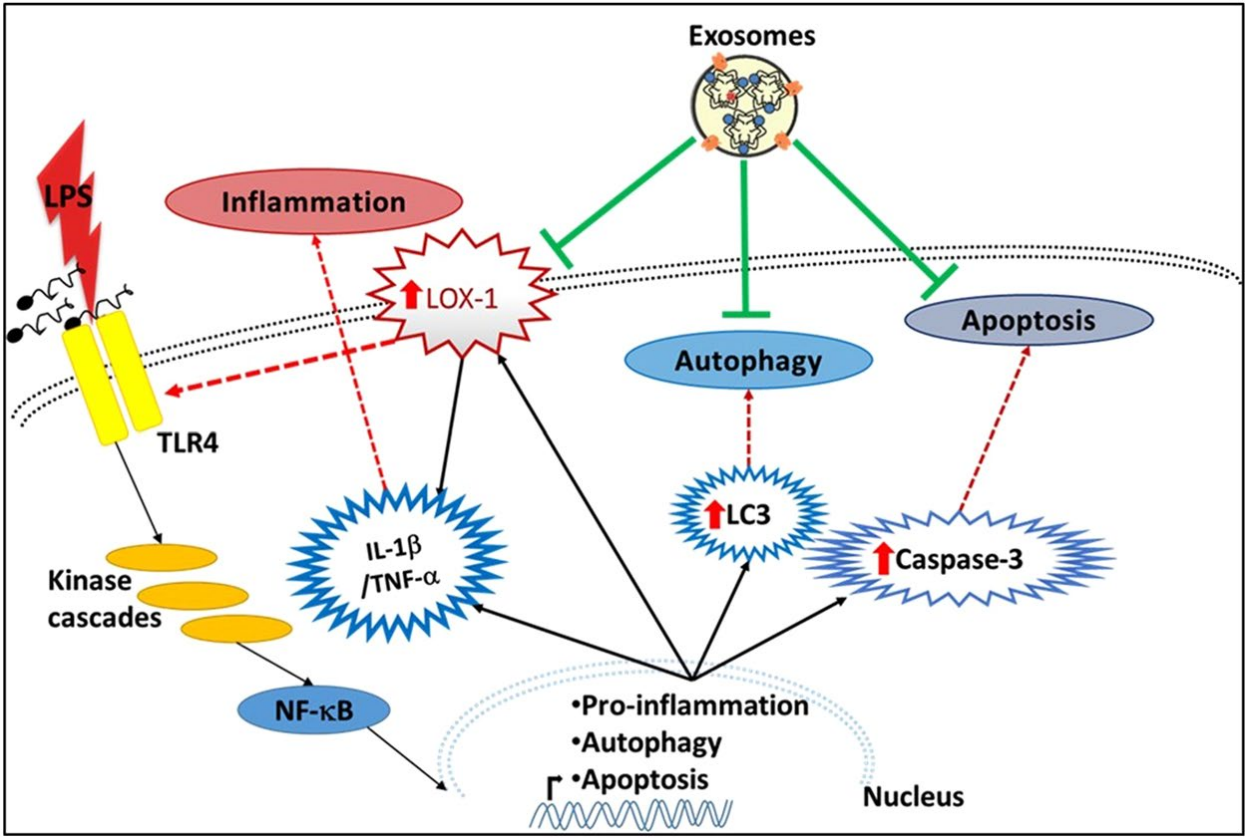
MSC Exosomes provide significant cardioprotection against LPS stimulated stress responses. LPS treatment significantly increases pro-inflammatory LOX-1 expression along with cleaved caspase-3, and LC3. In spite of presence of LPS and continued stimulation of TLR4 LPS receptors, addition of MSC exosomes reverses the effects of LPS by downregulating expression of LOX-1, caspase-3 and LC3, thereby decreasing apoptosis and autophagy.

We believe that use of exosomes from MSCs would positively modulate the ischemic microenvironment of cardiac tissues and help damaged cardiomyocytes recover, repair and reverse the consequence of the inflammatory event. We have shown that MSC exosomes suppress apoptosis and autophagy in LPS stimulated cardiomyocytes as well as LOX-1 expression. These effects may be consequence of exosome cargo (Fig. 8). Although many studies have attempted to identify exosomal microRNAs as early diagnostic biomarkers for various diseases^47–51^, we believe that it is the physiologically active exosomal protein content which ultimately governs the role exosomes play in modulating the recipient cellular physiology. In our future studies, we will look into the secretion of many of the transcription factors via exosomes and their roles in recipient cells.

Our study will lay the ground work to utilize stimulated stem cell/cardiomyocyte exosomes, to effectuate cardioprotection in damaged heart tissues.

## Methods

### Cell culture and reagents

Cell culture media, as previously described^34^– DMEM, phosphate buffered saline (PBS), penicillin/streptomycin were obtained from Corning (Thermo Fischer Scientific, Waltham, MA), fetal bovine serum (FBS) from Atlanta Biologicals Inc (Flowery Branch, GA). LPS was purchased from Sigma Aldrich (St. Louis, MO). MTS assay regent (celltiter 96 aqueous one solution cell proliferation assay) was purchased from Promega Corporation (Madison, WI). LDH cytotoxicity assay kit was purchased from Thermo scientific (Rockford, IL). The rat myoblast cell line, H9C2 (CRL-1446) was purchased from ATCC. Mesenchymal stromal cell (MSC) line were obtained from Robert J. Griffin, Ph.D. at UAMS.

### Cell culture

H9C2 cultures were maintained in complete DMEM media containing 10% FBS & 100 units/ml of penicillin/streptomycin at 37 °C in a 5% CO_2_ incubator. As per our previously developed protocol^35^, overnight cell cultures grown to 80% confluence were used for experiments. Cells cultured overnight were washed 3x with PBS and fresh serum free DMEM media was added and were then incubated for a further for indicated time points at 37 °C in a 5% CO_2_ incubator. For experiments, these cells were treated with LPS initially for 1 hour and then MSC exosomes were added to the culture media in presence of LPS. A small quantity of the lysates (50μg) was sued for MS analysis. For spheroid cultures, H9C2 cells were suspended in serum free DMEM medium and 20 μl of cell suspension was plated onto the covers of 6 well tissue culture plates to form hanging drops. The covers were then inverted and placed on the plates containing PBS to keep the cell suspension hydrated. Cells were allowed to form spheroids over a period of 2-3 days and then treated with exosomes alone or LPS or with LPS for 1 hour and then MSC exosomes were added without washing off the LPS.

MSCs maintained in complete DMEM media containing 10% FBS & 100 units/ml of penicillin/streptomycin at 37 °C in a 5% CO_2_ incubator. In brief^35^, cells cultured overnight were washed 3x with PBS and fresh serum free DMEM media was added and were then incubated for a further 16–18 hrs at 37 °C in a 5% CO_2_ incubator. Media from these cultures grown was collected to isolate exosomes. For hypoxic exosomes, MSCs were grown in an anaerobic chamber (Forma Scientific) with an atmospheric mixture of 5% CO_2_, 10% H_2_, 85% N_2_ producing oxygen concentrations below 0.5%.

### Exosome isolation

Exosomes were isolated from conditioned media of MSC cultures incubated in serum free DMEM media for 16–18 hrs by sequential centrifugation as previously described^34^. In brief, media pooled from MSC cultures was subjected to series of centrifugation steps, first at 3000 g for 10 min followed by centrifugation for 30 min at 10,000 g. The pellets were discarded and followed by ultracentrifugation at 100,000 g for 3 hrs to pull down exosomes. The resulting exosome pellet was resuspended in PBS and ultracentrifuged for 2 hrs at 100,000 g. The final pellet thus obtained was resuspended in PBS and used in further experiments.

### ZetaView Nanoparticle tracking analysis

Isolated exosomes were subjected to nanoparticle tracking analysis using the ZetaView PMX 110 (Particle Metrix, Meerbusch, Germany). In brief as described previously^52^, he sample was diluted in 1 ml of PBS (200 fold) and loaded into the optical cell. Using two reading cycles at each position, 11 different positions inside the cells were read by the instrument. Acquisition parameters were 23 °C temp., 90 sensitivity and a frame rate of 30 frames per second (fps), a shutter speed of 100, and a laser pulse set to the shutter speed. The mean, median and mode were calculated by the software (ZetaView 8.04.02 SP2) and outliers discarded. Post-acquisition parameters were set to a minimum brightness of 25, a maximum area of 1000 pixels, and a minimum area of 10 pixels. Polystyrene particles from ThermoFisher Scientific with a known average size of 100 nm were used to calibrate the instrument prior to sample readings. Automated quality control measurements including, cell quality check and instrument alignment and focus were also performed prior to the use of the ZetaView for sample measurements.

### Electron microscopy

Exosomes coated on glass surface were fixed overnight at 4 °C in 3% glutaraldehyde in 0.1 M cacoldylate buffer pH 7.2. Then Samples were washed three times with 0.1 M cacoldylate buffer pH 7.2 and three times with distilled water 10 min each, after that the samples were fixed in 4% osmium tetroxide in distilled water for 1 hour, and then the samples were rinsed again with 0.1 M cacoldylate buffer pH 7.2 and distilled water three times 10 min each. This was followed by dehydration process by using a graded series of ethanol with two final dehydration steps in 100% ethanol for 30 mins each. Samples were transferred to a 1:2 solution of hexamethyldisilazane (HMDS):100% ethanol for 20 minutes, then treated with a solution of 2:1 HMDS:ethanol for 20 minutes. Then the samples were submerged in 100% HMDS solution in a loosely capped container in a fume hood overnight to remove the HMDS. Finally, the samples were mounted over STUBS and then coated with carbon layers to enhance the conductivity. Images were acquired using the JEOL JSM7000F scanning electron microscope.

### Cell death and viability assays

Cell viability assay was performed following the instructions published by the manufacturer of the MTS reagent. In brief, as previously performed^35^, H9C2 cells were seeded into 96 well plate at a density of 3000 cells per well and allowed to attach overnight (18 hrs). Fresh exosome free media was added to the cells after a wash with PBS, the cells were treated with 50 nM of lipopolysaccharide (LPS) for 1 hr and then incubated for 24 or 48 hrs with or without exosomes, in presence of LPS in a humidified, 5% CO_2_ atmosphere. At indicated time points, 50 μl of media from each well was collected and added into fresh wells and to the remainder 20 μl of MTS regent was added. To develop the reaction, the cells were incubated for 4 hrs after which absorbance at 570 nm was read in a 96-well plate reader. To the 50 μl of collected media in clean wells, 50 μl of LDH reagent was added and incubated for 30 mins at room temp. The reaction was quenched with addition of 50 μl of stop buffer and absorbance read at 490 and 680 nm in a 96 well plate reader.

### SDS-PAGE and western blotting

Using a widely employed and previously used protocol to develop Westerns blots^34,53^, around 20 μg of exosomal pellets or cell lysates, determined by BCA protein estimation, were boiled with 4x Laemmle’s sample buffer containing 10% beta-mercaptoethanol and run on a 4–20% SDS-PAGE denaturing gel (Bio-Rad Laboratories, Hercules, CA). Using conventional transfer apparatus, proteins from the gel were transferred to PVDF membranes. Either 5% Bovine serum albumin (BSA) or 5% non-fat dried skimmed milk powder dissolved in Tris-buffered saline containing 0.01% tween-20 (TBS-t) was used to block the membranes for 1 hr at room temp. Blots were incubated with primary antibodies against caspase-3 (rabbit polyclonal antibody at 1:1000 dilution), LOX-1 (rabbit polyclonal antibody at1:1000 dilution), Beclin-1 (rabbit polyclonal antibody at 1:1000 dilution), LC-3 (rabbit polyclonal antibody at 1:1000 dilution), (all from Abcam, Cambridge, MA), CD63 (rabbit polyclonal antibody at 1:1000 dilution) (SBI Biosciences, Mountain View, CA) and against β-actin (mouse monoclonal antibody at 1:5000 dilution) (Abcam, Cambridge, MA)] for 1 hr at room temp. Blots were washed 3x with TBS-t and then incubated with anti-mouse or anti- rabbit antibody conjugated with horse radish peroxide (HRP) (at 1:10,000 dilution) (Santa Cruz Biotech, Dallas, TX) at room temperature for 1 hour. Blots were rinsed 3x with TBS-t and developed with a chemiluminescence developing reagent^34^, SuperSignal® West Femto Maximum sensitivity substrate (ThermoFisher Scientific, Rockford, IL).

### Mass spectrometry

FASP Methods – Orbitrap Lumos: LC-MS/MS methods – Orbitrap Velos: Purified proteins were reduced, alkylated, and digested using filter-aided sample preparation. Tryptic peptides were then separated by reverse phase XSelect CSH C18 2.5 um resin (Waters) on an in-line 150 × 0.075 mm column using an UltiMate 3000 RSLCnano system (Thermo). Peptides were eluted using a 90 min gradient from 97:3 to 60:40 buffer A:B ratio, [Buffer A = 0.1% formic acid, 0.5% acetonitrile; buffer B = 0.1% formic acid, 99.9% acetonitrile]. Eluted peptides were ionized by electrospray (2.15 kV) followed by MS/MS analysis using higher-energy collisional dissociation (HCD) on an Orbitrap Fusion Lumos mass spectrometer (Thermo) in top-speed data-dependent mode. MS data were acquired using the FTMS analyzer in profile mode at a resolution of 240,000 over a range of 375 to 1500 m/z. Following HCD activation, MS/MS data were acquired using the ion trap analyzer in centroid mode and normal mass range with precursor mass-dependent normalized collision energy between 28.0 and 31.0. Proteins were identified by database search using MaxQuant (Max Planck Institute) with a 3 ppm parent ion tolerance and a 0.5 Da fragment ion tolerance. Peptide and protein identifications were verified using Scaffold 4 software. Protein identifications were accepted if they could be established with less than 1.0% false discovery and contained at least 2 identified peptides. Protein Prophet Algorithm^54^ was used to assign protein probabilities. The Mascot results were viewed and analyzed by Scaffold Q + (version Scaffold_4.8.9, Proteome Software Inc., Portland, OR). A protein threshold of 95%, minimum of 2 peptides and a 50% peptide threshold values were used as the cut-off values and resulting data exported into excel spreadsheets.

### Statistical analysis

As previously described^34,35,53^, we used GraphPad Prism’s two way ANOVA followed by Bonferroni’s multiple comparison test to calculate statistical significance for all results, expressed as mean ± SD. Value of p < 0.05 was considered statistically significant,

## Supplementary information

Supplementary Figures

MSC exosome proteomics dataset 1
