# Author Correction: Molecular events in MSC exosome mediated cytoprotection in cardiomyocytes

**DOI:** 10.1038/s41598-022-11690-y

**Published:** 2022-05-12

**Authors:** Rajshekhar A. Kore, Jeffrey C. Henson, Rabab N. Hamzah, Robert J. Griffin, Alan J. Tackett, Zufeng Ding, Jawahar L. Mehta

**Affiliations:** 1 Department of Medicine, Cardiology Division, University of Arkansas for Medical Sciences, Little Rock, AR and the Central Arkansas Veterans Healthcare System, Little Rock, AR 72205 USA; 2 Department of Radiation Oncology, University of Arkansas for Medical Sciences, Little Rock, AR 72205 USA; 3 Department of Biochemistry and Molecular Biology, University of Arkansas for Medical Sciences, Little Rock, AR 72205 USA; 4 Center for Integrative Nanotechnology Sciences, University of Arkansas at Little Rock, Little Rock, AR 72204 USA

Correction to: *Scientific Reports* 10.1038/s41598-019-55694-7, published online 17 December 2019

The original version of this Article contained an error in Figure 2, where the western blot image for cleaved Caspase-3 for the 12 hr time point was incorrect. The original Figure 2 appears below.

**Figure 2:**
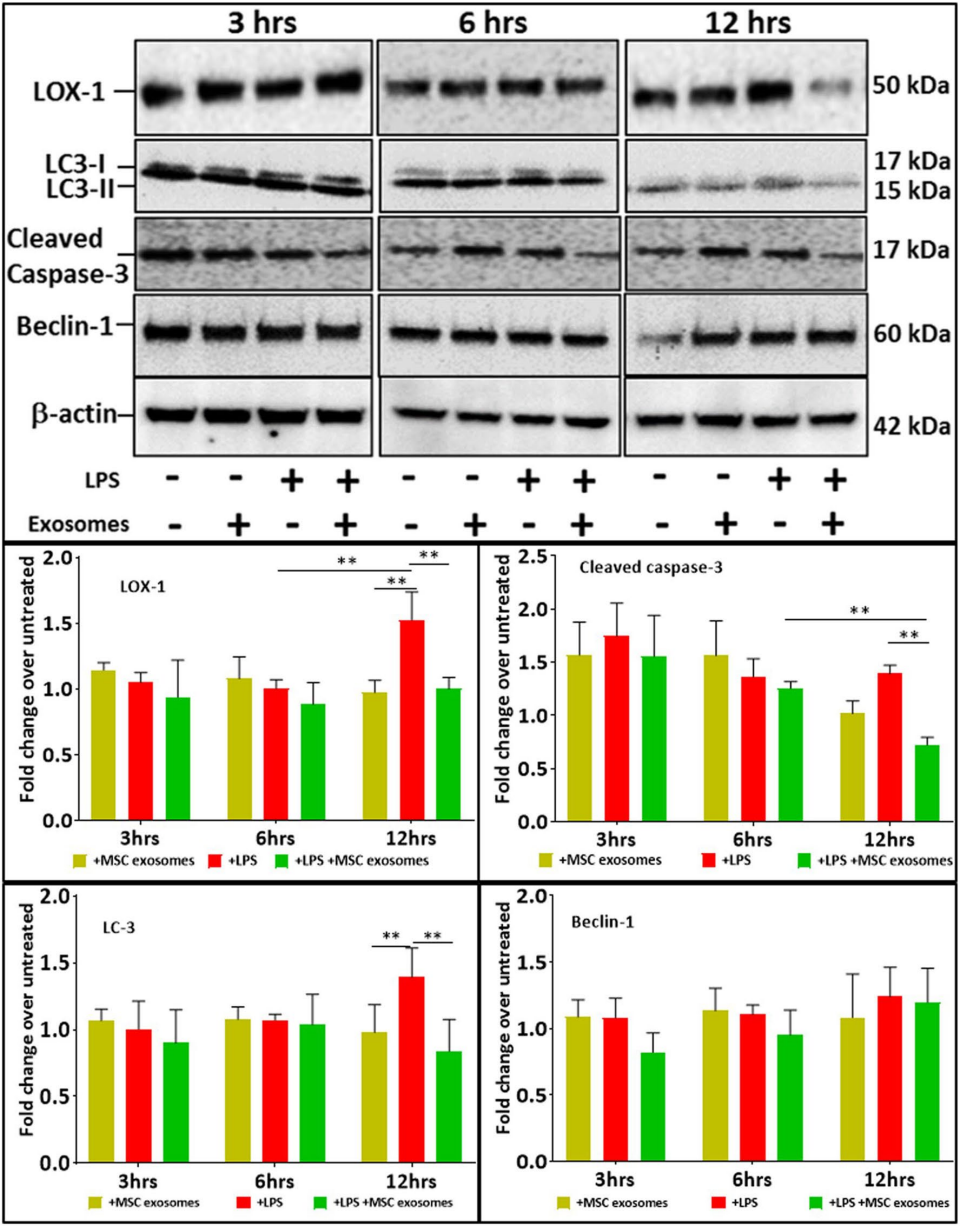
Monolayer cultures of cardiomyocytes were exposed to LPS (100 ng/ml) for 1 hour and then treated with MSC exosomes. LPS induced LOX-1 expression at 12 hrs of exposure. LPS also induced apoptosis (cleaved Caspase-3) and autophagy (LC3). Treatment with MSC exosomes decreased LOX-1, cleaved caspase-3 and LC-3 levels in cardiomyocytes stressed with LPS over a period of 6–12 hrs, but had no effect on expression of Beclin-1. LOX-1 levels were decreased with MSC exosomes. Data in mean ± SD, n = 4 *p < 0.05, vs LPS treatment based on 3 independent experiments. Full-length blots are presented in Supplementary Fig. 2.

Consequently, the Supplementary Figures file published with this Article contained an error in Supplementary Figure 2, where the full-length western blot image for cleaved Caspase-3 for the 12 hr time point was incorrect. The original Supplementary Figures file is provided below.

These errors have now been corrected in the original Article and in the Supplementary Figures file that accompanies the original Article.

## Supplementary Information

Supplementary Figures.

