# Supplementary material for: Molecular events in MSC exosome mediated cytoprotection in cardiomyocytes
Source: Sci Rep. 2019 Dec 17;9:19276. doi: 10.1038/s41598-019-55694-7 (PMC6917778; doi:10.1038/s41598-019-55694-7)
Supplement: Supplementary file 1 — Supplementary Figures [file 41598_2019_55694_MOESM1_ESM.docx]

**Molecular events in MSC exosome mediated cytoprotection in cardiomyocytes**

Rajshekhar A. Kore^1^, Jeffrey C Henson^1^, Rabab N. Hamzah^2,4^, Robert J. Griffin^2^, Alan J. Tackett^3^, Zufeng Ding^1^, Jawahar L. Mehta^1‡^

^1^Department of Internal Medicine, Cardiology Division, University of Arkansas for Medical Sciences, Little Rock, AR 72205, USA.

^2^Department of Radiation Oncology, University of Arkansas for Medical Sciences, Little Rock, AR 72205, USA.

^3^Department of Biochemistry and Molecular biology, University of Arkansas for Medical Sciences, Little Rock, AR 72205, USA.

^4^Center for Integrative Nanotechnology Sciences, University of Arkansas at Little Rock, Little Rock, AR 72204, USA.

‡ Corresponding author Address: Department of Internal Medicine, Cardiology division, University of Arkansas for Medical Sciences, 4301 W. Markham St., Slot 532, Little Rock, AR 72205, USA. Email: [MehtaJL@uams.edu](mailto:MehtaJL@uams.edu); Fax: +1 501 686 6180; Phone: +1 501 257 5558

Supplementary Figure 1:


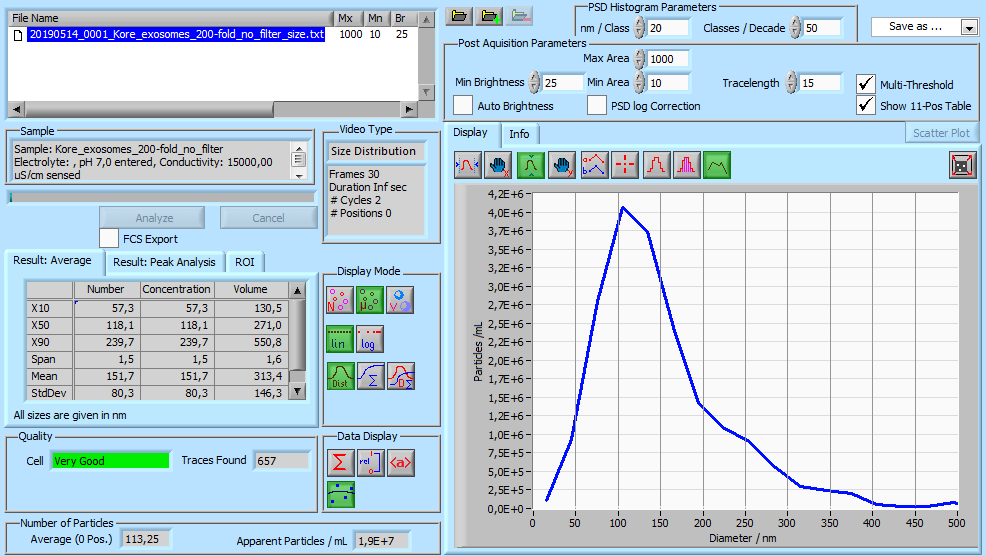


*ZetaView measurements of MSC exosomes isolated from MSCs cultured in serum free medium. ZetaView instrument was used to carry out the NTA analysis to measure exosome vesicle diameters (nm)*

Supplementary Figure 2:


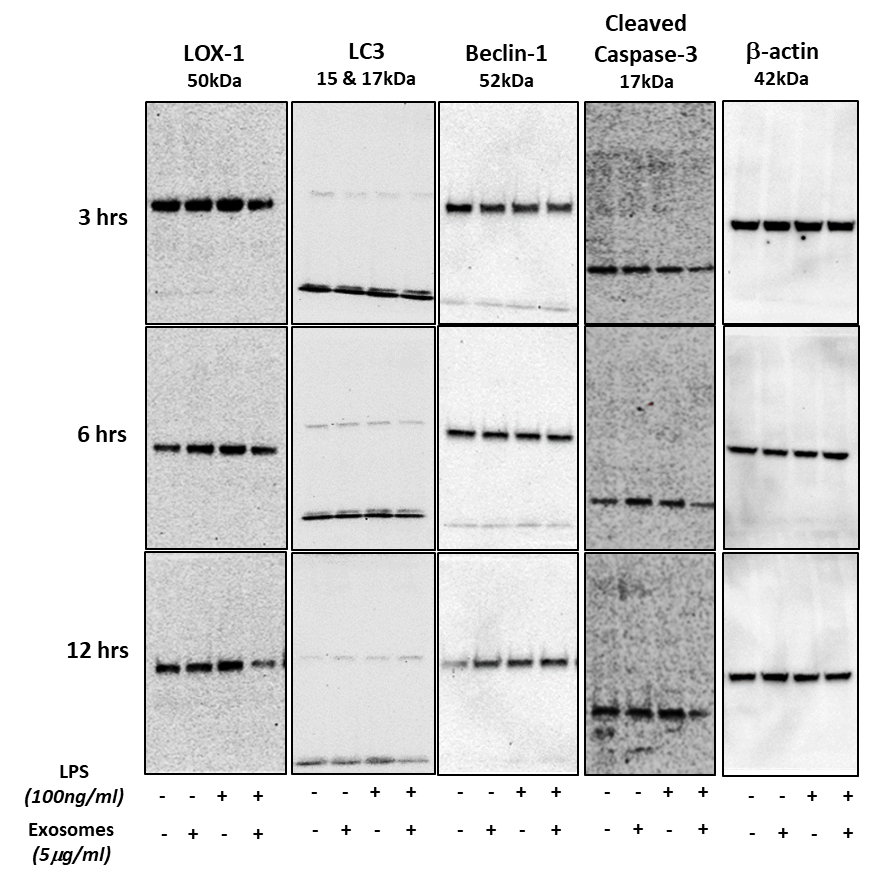


*Monolayer cultures of cardiomyocytes were exposed to LPS (100 ng/ml) for 1 hour and then treated with MSC exosomes (in presence of LPS). Full-length blots are presented. The images were acquired with the BioRad Molecular Imager™ ChemiDoc® XRS+. The images were then processed Image Lab software ™ and the brightness and contrast were manipulated to present the blots. Images were copied and pasted onto powerpoint slides and processed for brightness and contrast.*

Supplementary Figure 3:

***(A)***


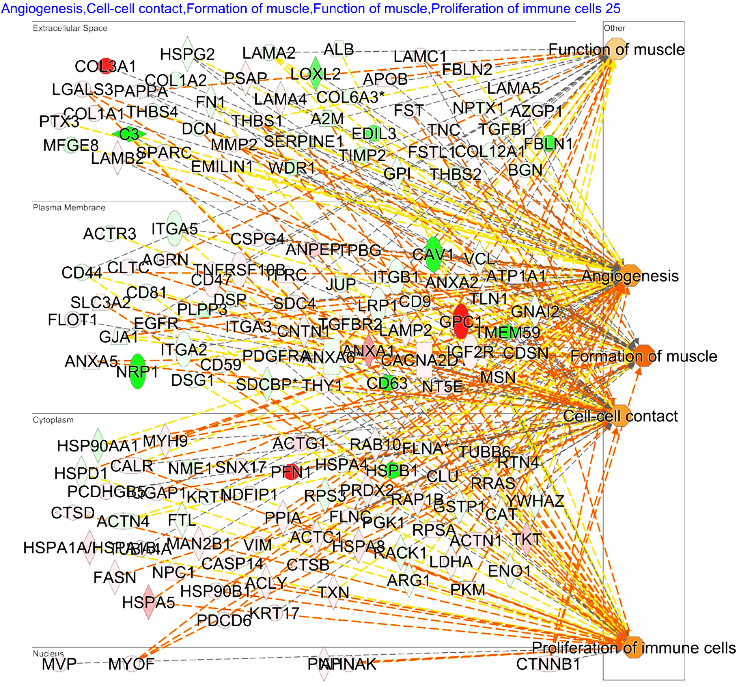


***(B)***


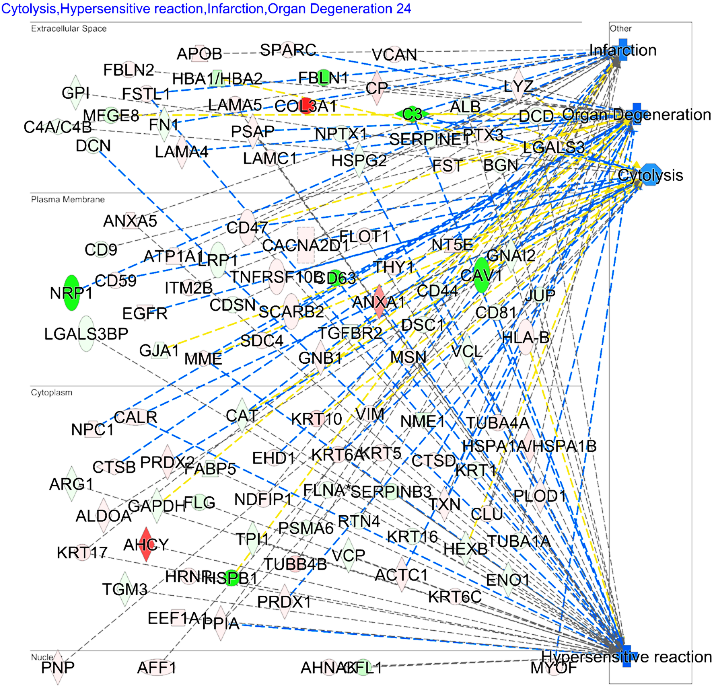


*Network analysis of MSC exosomal proteome. Ingenuity pathway analysis (QIAGEN Inc., https://www.qiagenbioinformatics.com/products/ingenuity-pathway-analysis)^18^ of 338 proteins identified in MSC exosomes shows cellular components, interactive partners and important pathways that these proteins are potentially involved in. Orange nodes (A) indicate pathways that are upregulated while blue nodes show downregulated pathways (B).*

Supplementary Figure 4:

| **Molecules** | **Expr. Value**  ***(B) Top downregulated proteins***  ***(C)*** |
| --- | --- |
| PSME4 | -3.3298 |
| ACADVL | -3.2251 |
| FAM91A1 | -3.1722 |
| GNA11 | -3.0689 |
| STAT6 | -3.0676 |
| MCU | -2.5563 |
| CACYBP | -2.3899 |
| DNPEP | -2.1646 |
| PSMB2 | -2.1542 |
| CHERP | -1.9517 |

| **Molecules**  ***(C) Top upregulated proteins***  ***(C)*** | **Expr. Value** |
| --- | --- |
| NCBP1 | 3.7354 |
| WBP11 | 3.5214 |
| ACAT2 | 3.2159 |
| FXR2 | 2.3823 |
| SPART | 2.3104 |
| ELOB | 2.1406 |
| AHNAK2 | 2.0635 |
| SNX3 | 2.0343 |
| STARD9 | 1.9929 |
| NMT1 | 1.8164 |


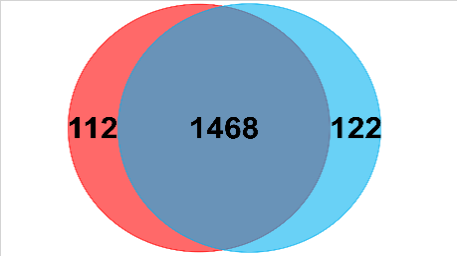


***LPS stimulated CM***

***LPS stimulated CM***

***Untreated CM***

***Untreated CM***

***(A)***

***(D)***

***(D)***


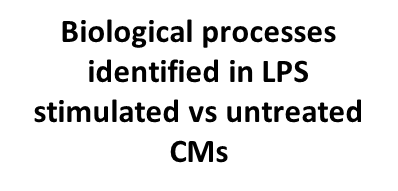

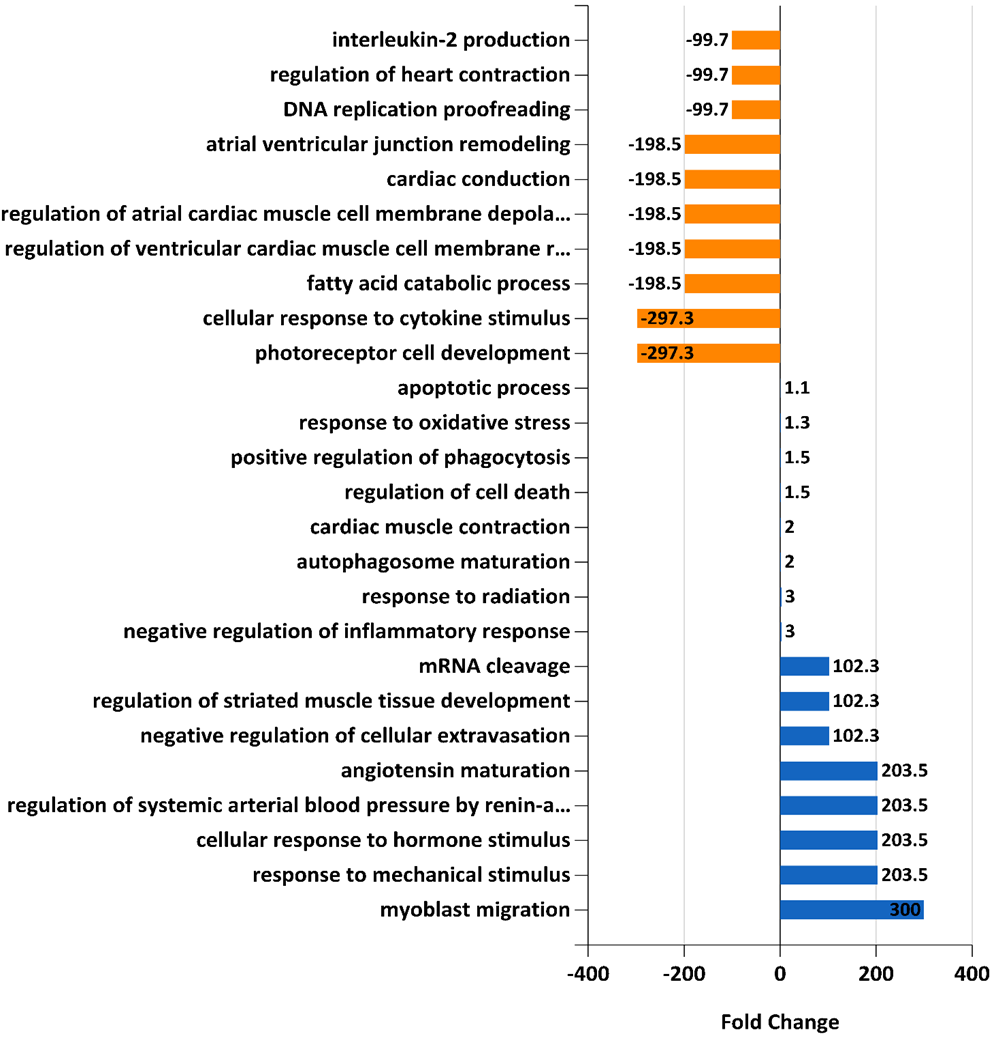


*MS analysis of LPS stimulated cardiomyocyte (CM) proteome. MS identified 1590 proteins in untreated CMs (blue) while 1580 proteins were identified in LPS stimulated CMs (red). Comparative analysis of proteins using FunRich^17^ analysis tool show differential expression ((B) green=downregulated; (C) red=upregulated) of many proteins in LPS stimulated CMs. (D) Gene enrichment analysis shows differential regulation of biological processes by proteins identified in LPS stimulated CMs.*

Supplementary Figure 5:

***(B) Top downregulated proteins***

***(C)***

| **Molecules**  ***(C) Top upregulated proteins***  ***(C)*** | **Expr. Value** |
| --- | --- |
| GIT2 | -3.2585 |
| GIT1 | -2.6475 |
| VPS29 | -2.3459 |
| DNAJA2 | -2.2985 |
| RAB8A | -2.2332 |
| FKBP4 | -2.0287 |
| TECR | -1.9985 |
| DCTN4 | -1.8852 |
| USP15 | -1.8633 |
| ACAD9 | -1.7375 |

| **Molecules** | **Expr. Value** |
| --- | --- |
| CDK1 | 3.7559 |
| MACF1 | 3.6599 |
| ACAT2 | 3.6138 |
| CAND2 | 3.0909 |
| UBE2D3 | 2.9261 |
| WBP11 | 2.773 |
| ELOB | 2.7328 |
| DDX23 | 2.7052 |
| NFIC | 2.4297 |
| SPART | 2.3597 |


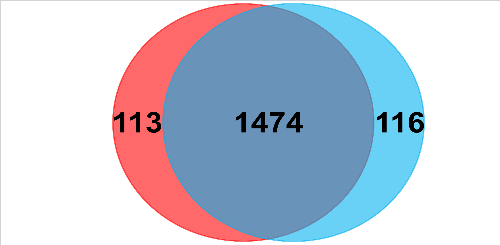


***LPS stimulated, MSC exosome rescued CM***

***LPS stimulated, MSC exosome rescued CM***

***Untreated CM***

***Untreated CM***

***(A)***

***(A)***

*MS analysis of MSC exosome treated LPS stimulated cardiomyocyte (CM) proteome. Compared to 1590 identified proteins in untreated CMs (blue) 1587 proteins were identified MSC exosome treated LPS stimulated CMs (red). Comparative analysis of proteins using FunRich^17^ analysis tool show differential expression ((B) green=down regulated; (C) red=upregulated) of many proteins in LPS stimulated CMs.*

Supplementary Figure 6:

***(B)***

***(B)***

***(C)***

***(C)***


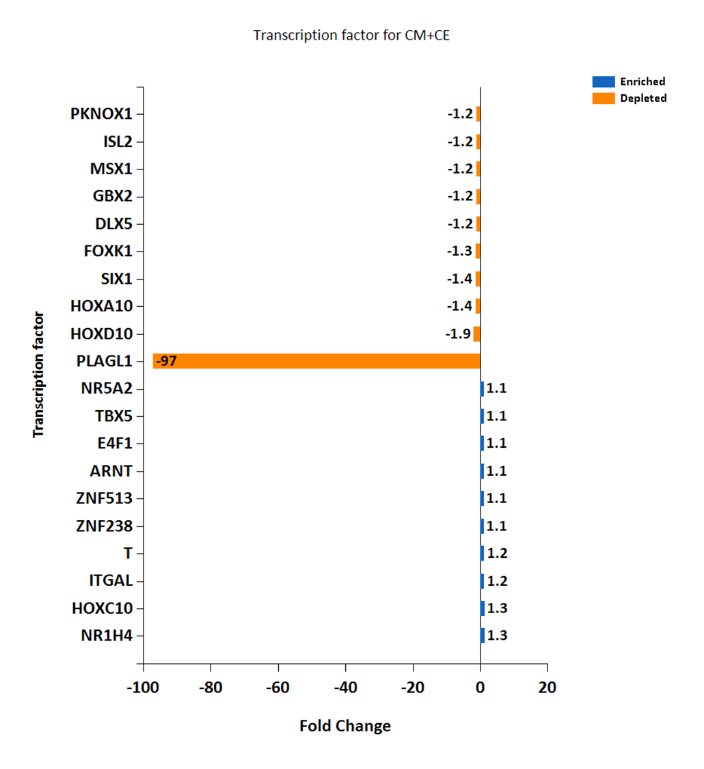

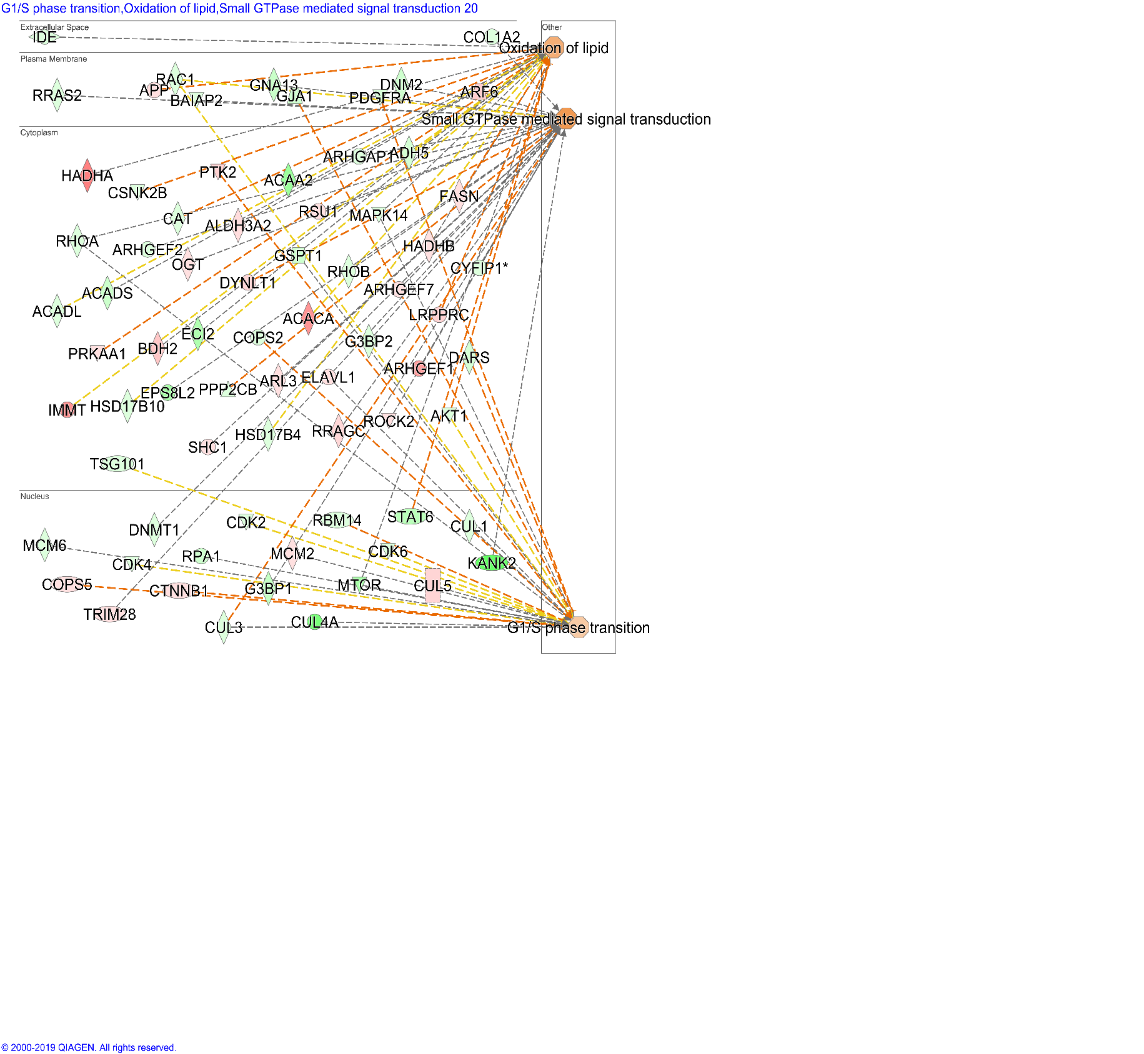

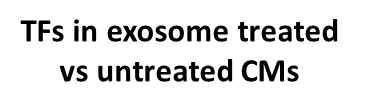


***(A)***

***(A)***


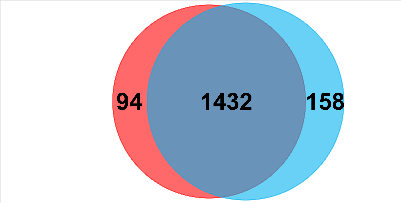


***MSC exosome treated CM***

***MSC exosome rescued CM***

***Untreated CM***

***Untreated CM***


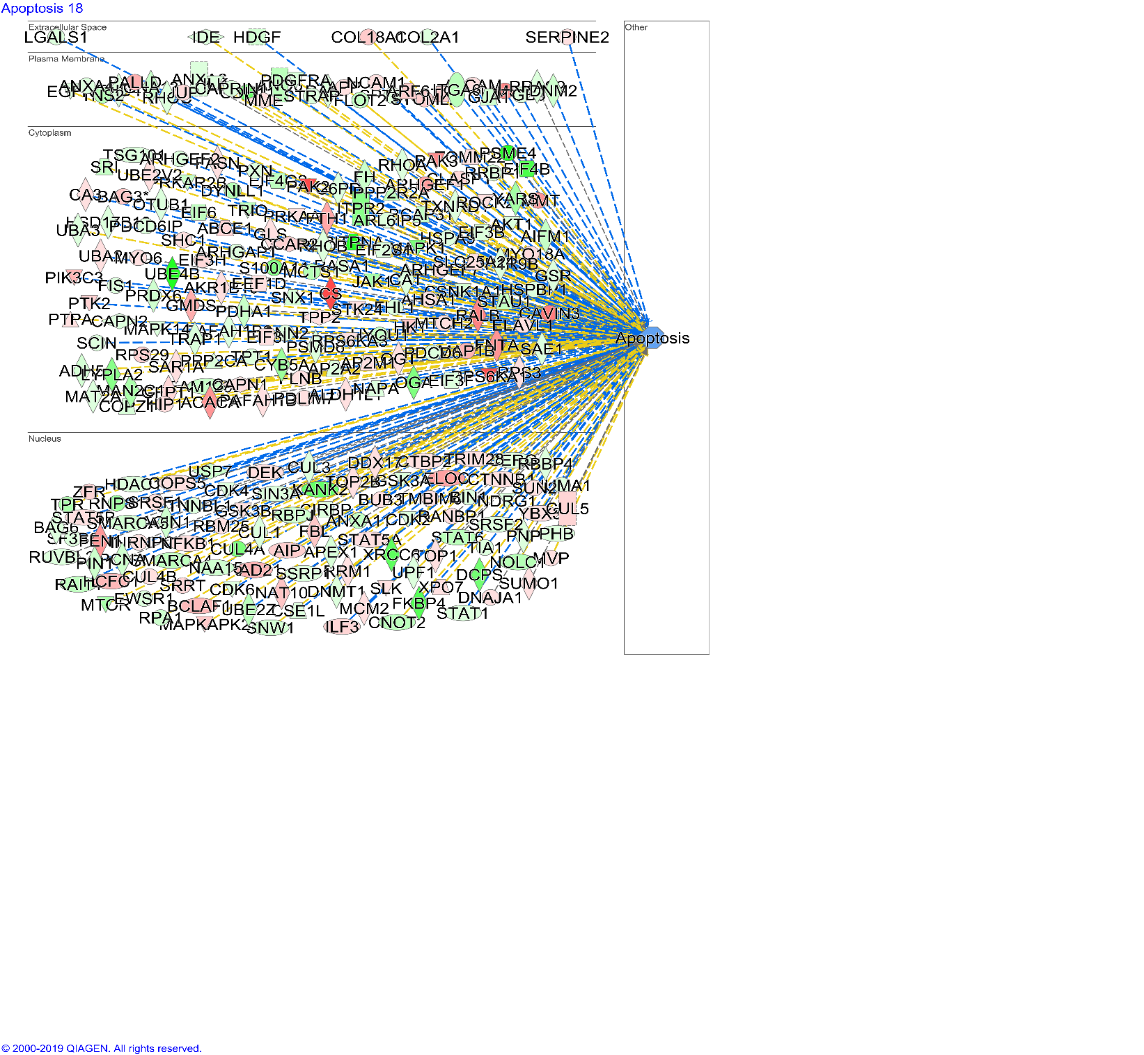

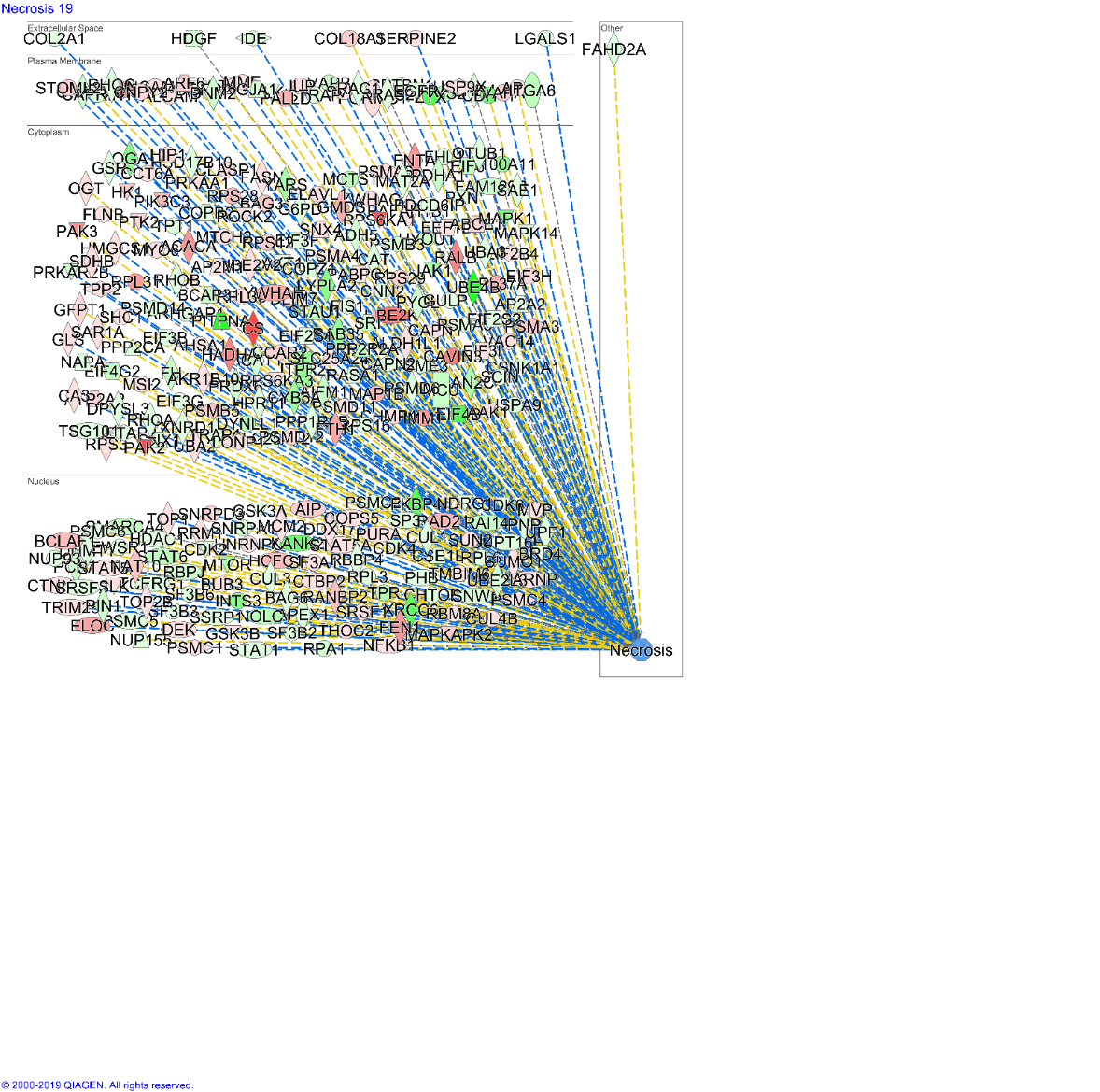


***(D)***

***(D)***

***(E)***

***(E)***

*MS and Network analysis of MSC exosome treated cardiomyocyte (CM) proteome. Compared to 1590 proteins identified in untreated CMs (blue) 1526 proteins were identified in MSC exosome treated CMs (red). Comparative analysis using FunRich analysis^17^ of TFs show that PLAGL1 was the major TF substantially downregulated in exosome treated CMs (B). Ingenuity pathway analysis (QIAGEN Inc.,* [*https://www.qiagenbioinformatics.com/products/ingenuity-pathway-analysis*](https://www.qiagenbioinformatics.com/products/ingenuity-pathway-analysis)*)^18^ of 338 proteins secreted by MSCs shows their cellular localization, their interactive partners and the various biological functions that these proteins are potentially involved in. Nodes colored orange (C) were upregulated pathways while nodes colored blue, were downregulated pathways especially modulating necrosis (D) and apoptosis (E).*


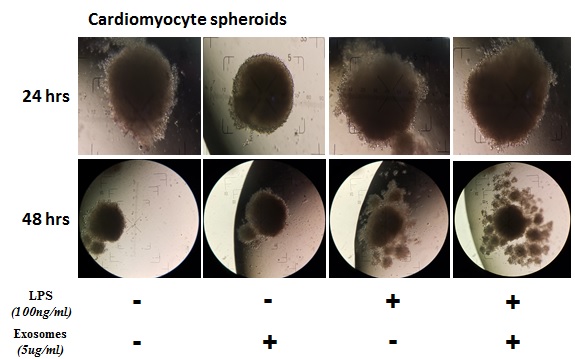
Supplementary Figure 7:

*Representative images of the cardiomyocyte (H9C2) spheroids generated by the hanging drop method.*
